# Neuroprotection by Epigenetic Modulation in a Transgenic Model of Multiple System Atrophy

**DOI:** 10.1007/s13311-016-0447-1

**Published:** 2016-06-03

**Authors:** Edith Sturm, Lisa Fellner, Florian Krismer, Werner Poewe, Gregor K. Wenning, Nadia Stefanova

**Affiliations:** Division of Neurobiology, Department of Neurology, Medical University of Innsbruck, Anichstr. 35, 6020 Innsbruck, Austria

**Keywords:** α-Synuclein, nigral degeneration, phenylbutyrate, histone acetylation, neuroprotection

## Abstract

Similar to Parkinson disease, multiple system atrophy (MSA) presents neuropathologically with nigral neuronal loss; however, the hallmark intracellular α-synuclein (αSyn) accumulation in MSA affects typically oligodendrocytes to form glial cytoplasmic inclusions. The underlying pathogenic mechanisms remain unclear. As MSA is predominantly sporadic, epigenetic mechanisms may play a role. We tested the effects of the pan-histone deacetylase inhibitor (HDACi) sodium phenylbutyrate in aged mice overexpressing αSyn under the control of the proteolipid protein promoter (PLP–αSyn) designed to model MSA and characterized by αSyn accumulation in oligodendrocytes and nigral neurodegeneration. HDACi improved motor behavior and survival of nigral neurons in PLP–αSyn mice. Furthermore, HDACi reduced the density of oligodendroglial αSyn aggregates, which correlated with the survival of nigral neurons in PLP–αSyn mice. For the first time, we suggest a role of HDACi in the pathogenesis of MSA-like neurodegeneration and support the future development of selective HDACi for MSA therapy.

**Electronic supplementary material:**

The online version of this article (doi:10.1007/s13311-016-0447-1) contains supplementary material, which is available to authorized users.

## Introduction

Recently, histone acetylation has been implicated in the pathogenesis of various neurodegenerative diseases [1–3]. Histone acetyltransferases and histone deacetylases (HDACs) mediate histone acetylation and deacetylation. They support chromatin stability and provide an important epigenetic mechanism for modulation of gene transcription, which may play a role in the pathogenesis of predominantly non-genetic disorders. Multiple system atrophy (MSA) is a sporadic neurodegenerative disease that includes autonomic failure, parkinsonism, and cerebellar ataxia, in any combination, reflecting multisystem neuronal loss, gliosis, and α-synuclein (αSyn) pathology [4, 5]. αSyn-positive glial cytoplasmic inclusions (GCIs) represent the pathological hallmark of MSA and classify MSA as one of the α-synucleinopathies, together with Parkinson’s disease (PD) and dementia with Lewy bodies [6]. The location of αSyn inclusions in MSA brains is not limited to the oligodendroglial cytoplasm but can be found in nuclear inclusions in neuronal, as well as in glial cells [7, 8]. αSyn interacts with histones in the nucleus, leading to inhibition of acetylation and finally resulting in neurotoxicity [9]. Therefore, we hypothesize that αSyn in MSA may interfere with histone acetylation in glial and neuronal cells and that this may play a role in the disease process, thus representing a new potential therapeutic target. To test this hypothesis, we sought to analyze the effects of sodium phenylbutyrate (NaPB), a nonselective pan-HDAC inhibitor (HDACi) in a transgenic mouse model of MSA, based on targeted overexpression of full-length human αSyn under the proteolipid protein (PLP) promoter in oligodendrocytes [10].

## Materials and Methods

### Animals and Drug Treatment

We housed MSA transgenic mice with targeted overexpression of human αSyn under the PLP promoter (henceforth referred to as PLP–αSyn mice and previously described [10]), as well as nontransgenic sex- and age-matched controls of the background strain C57Bl/6 in a temperature-controlled room under a 12-h light/dark cycle. Mice had free access to water and food at the specific pathogen-free animal facility of the Medical University Innsbruck. We undertook all efforts possible to minimize the number and suffering of animals used. We performed all animal procedures according to Austrian Law and approved by the Federal Ministry of Science and Research, Austria. We genotyped mice by ear punching followed by DNA isolation and polymerase chain reaction for human αSyn with specific primers (forward: 5’-ATG GAT GTA TTC ATG AAA GG-3’; reverse: 5’-TTA GGC TTC AGG TTC GTA G-3’) resulting in a 450-base pair polymerase chain reaction product. We randomized transgenic, as well as nontransgenic controls of the background strain C57Bl/6, mice at the age of 9 months, based on their weight, into 2 treatment groups. Treatment consisted of daily intraperitoneal injections of either 200 mg/kg body weight of NaPB (Santa Cruz Biotechnology, Heidelberg, Germany) in saline or vehicle only over 8 weeks. The dose and duration of treatment were based on data from previous experimental studies reporting neuroprotection by NaPB [11].

**Fig. 1:**
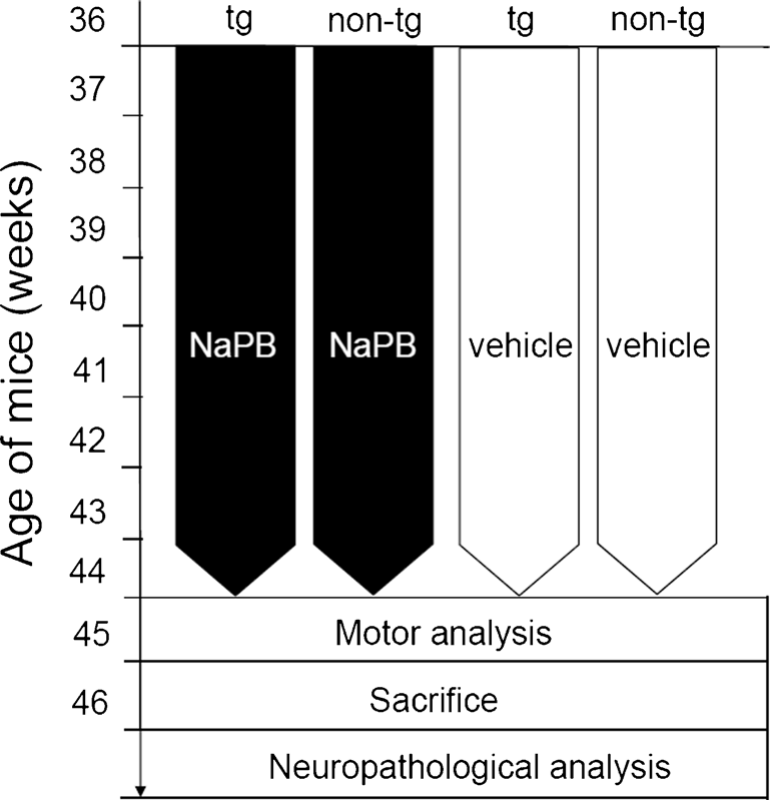
Experimental design and flowchart. tg = PLP –αSyn mice; non-tg = C57Bl/6 mice; NaPB = sodium phenylbutyrate

To assess the effects of the interventions, we assessed by behavioral tests the motor abilities of the mice. Immunoblotting and immunohistochemistry were performed to analyze the neuropathological status of substantia nigra pars compacta (SNc) after treatment, as described in the following protocols (Fig. 1).

### Gait Analysis

We performed motor behavior analysis immediately after completing daily NaPB/vehicle treatment (week 9). We used the DigiGait Analysis System (Mouse Specifics, Quincy, MA, USA) to measure the stride length of the animals. We placed the mice on a motorized transparent treadmill belt and recorded the animals’ gait at a speed of 25 cm/s from underneath, generating digital paw prints that were analyzed with the specific DigiGait Software 9.0 (Mouse Specifics), resulting in the mean stride length of each mouse.

### Chemicals and Antibodies

We obtained paraformaldehyde, phosphate-buffered saline (PBS), sucrose, H_2_O_2_, Trizma hydrochloride Ultra, trichloroacetic acid, acetone, bicinchoninic acid solution, copper (II) sulfate-pentahydrate, Ponceau-Red, Tween-20, sodium dodecyl sulfate, 2-β-mercaptoethanol, 3,3’-diaminobenzidine benzidine tetrahydrochloride, bovine serum albumin (BSA), and cresyl violet acetate from Sigma-Aldrich (Vienna, Austria). NaOH pellets, 2-methylbutan Uvasol for Spectroscopy, Entellan, ethylenediaminetetraacetic acid, Triton X-100, and acetic acid were from Merck (Darmstadt, Germany). Bis-Tris (10%) gels, 10× NuPAGE Sample Reducing Agent, 4× NuPAGE LDS Sample Buffer, 20× NuPAGE SDS MES Running Buffer, NuPAGE Antioxidant, SeeBlue Pre-stained Protein Standard, MagicMark XP Western Protein Standard, and 20× NuPAGE Transfer Buffer were obtained from Novex Life Technologies (formerly Invitrogen, San Diego, CA, USA). NaOH and ethanol were from VWR (Radnor, PA, USA), 37% hydrochloric acid, n-butylacetate, NaCl, and 96% sulfuric acid were obtained from Roth (Karlsruhe, Germany). Thiopental was obtained from Sandoz (Kundl, Austria); methanol was from Fisher Scientific (Vienna, Austria); Amersham ECL Prime Blocking Agent and nitrocellulose membranes from GE Healthcare Bio-Sciences AB (Uppsala, Sweden); Western Bright Quantum from Advansta (Menlo Park, CA, USA), deoxycholic acid sodium salt from Fluka (Buchs, Switzerland); and Complete ULTRA Tablets (proteinase inhibitor cocktail with phenylmethylsulfonyl fluoride) was from Roche (Basel, Switzerland). Normal goat and horse serum was purchased from Gibco Life Technologies (San Diego, CA, USA).

We purchased polyclonal rabbit anti-DJ1/PARK7 (1:2000), polyclonal rabbit anti-acetyl-histone H3 (acH3; Lys9/18, 1:10,000), polyclonal rabbit anti-histone H3.3 (H3; 1:10,000), and polyclonal rabbit anti-acetyl-histone H4 (acH4; 1:10,000) from Millipore (Temecula, CA, USA). Monoclonal mouse anti-acetylated tubulin (1:4000) was from Sigma-Aldrich (St. Louis, MO, USA) and monoclonal mouse anti-α-tubulin (1:500) from Abcam (Cambridge, UK). Amersham ECL donkey anti-rabbit IgG horseradish peroxidase (HRP)-linked whole antibody NA934 and Amersham ECL sheep anti-mouse IgG HRP-linked whole antibody NA931 (1:10,000) were from GE Healthcare Bio-Sciences AB. Immunohistochemistry involved the use of monoclonal mouse anti-tyrosine hydroxylase clone TH-16 antibody (1:1000) from Sigma-Aldrich, monoclonal anti-human-αSyn 15G7 (1:200) from Enzo (Lörrach, Germany), and biotinylated anti-mouse IgG (H+L) and biotinylated anti-rat IgG (H+L) from Vector Laboratories (Burlingame, CA, USA).

### Tissue Preparation

For immunohistochemistry, we perfusion-fixed the animals under deep thiopental anesthesia (120 mg/kg body weight) with 0.1 M PBS for 4 min followed by ice-cold 4% paraformaldehyde in 0.1 M PBS for 14 min. We dissected the brains rapidly and postfixed them overnight at 4°C in the same fixative.

For cryoconservation, we washed brains in 0.1 M PBS, transferred to 30% sucrose in 0.1 M PBS until they sank, and froze them in 2-methylbutan (−50°C). For long-term storage, brains were maintained at −80°C.

For biochemical analysis, animals were perfused under deep thiopental anesthesia with 15–20 ml PBS. Brains were dissected, quickly frozen in liquid nitrogen, and stored at −80°C. For radioimmunoprecipitation assay (RIPA) lysate production, brain samples were homogenized with RIPA buffer (150 mM NaCl, 50 mM Tris–HCl, 1% deoxycholic acid, 1% Triton X-100, 1 mM ethylenediaminetetraacetic acid; pH 7.4) containing proteinase inhibitor cocktail (Complete ULTRA tablet) for 1 min on ice by using IKA Ultra Turrax T8 at level 5. Homogenates were centrifuged for 15 min at 16,000 *g* (4°C). The supernatant representing RIPA fraction was stored at −80°C for further Western blot analyses. For histone fraction extraction, the pellet was quickly resuspended in 300 μl 0.2 M H_2_SO_4_, using the IKA Ultra Turrax T8 at level 5. The sample was allowed to incubate on a rotating wheel overnight at 4°C. Samples were centrifuged for 10 min at 16,000g (4°C), supernatant incubated for 30 min on ice with equal amount of 66% trichloroacetic acid, and centrifuged again, as described previously. The pellet was washed twice with ice-cold acetone and centrifuged for 5 min at 16,000 *g* (4°C). The pellet was let to dry for 20 min at room temperature, resuspended in Aqua dest. and stored at −80°C.

### Immunoblotting

Protein concentration of RIPA lysates was measured by the bicinchoninic acid protein assay and 20 μg of each sample were loaded on 10% Bis-Tris gels from Novex Life Technologies for electrophoretic separation. Proteins were electrotransferred to nitrocellulose membranes (GE Healthcare Bio-Sciences), blocked for 1 h with 2% Amersham ECL Prime Blocking Agent in PBS containing 0.05% Tween-20 and incubated with primary antibody overnight at 4°C followed by incubation for 1 h with the appropriate HRP-conjugated secondary antibody. Detection with enhanced chemoluminescence reagent was performed with the Fusion FX system for Western blot and gel imaging, and quantified with FUSION CAPT V16.09b software (Vilber Lourmat, Marne La Vallée, France). The expression level of α-tubulin was used for normalization of protein expression in RIPA extracts and H3 expression level in histone extracts was applied to normalize the expression values of acH3 and acH4.

### Immunohistochemistry

Frozen brains were sliced serially to 40-μm sections using a cryostat (Leica, Nussloch, Germany). A series was mounted on microscope slides and used for standard cresyl violet staining. Further section series followed standard protocol for free-floating immunohistochemical staining. In brief, they were incubated with 0.3% H_2_O_2_ in PBS to quench endogenous peroxidase activity, blocked in PBS containing 5% normal serum and 1% BSA, and incubated with primary antibody in PBS containing 1% normal serum and 1% BSA overnight at 4°C. This was followed by incubation with the appropriate biotinylated secondary antibody (1:200 in PBS containing 1% normal serum and 1% BSA, for 1.5 h) and Vectastain ABC reagent for 1 h. Stainings were developed with 3,3’-diaminobenzidine, mounted on gelatinated microscope slides, dehydrated, and coverslipped with Entellan. Image analysis was performed by using a light microscope (Nikon E-800 microscope) and a computer-assisted image analysis system (Stereo Investigator Software; MicroBrightField Europe e.K., Magdeburg, Germany). The analyses included stereological counting of TH-positive or Nissl-stained neurons and determining the density of 15G7-positive aggregates per mm^2^ in SNc.

### Statistical Analysis

Statistical analyses were performed using GraphPad Prism 5.0 (GraphPad Inc., La Jolla, CA, USA). If not indicated otherwise, 2-way ANOVA and post-hoc Bonferroni’s correction for multiple comparisons was applied when comparing two independent factors (genotype and treatment). All data are represented as mean ± SEM, indicated in each figure. A *p*-value < 0.05 was set to determine statistical significance.

## Results

### NaPB Treatment Improves Motor Performance and Survival of Nigral Neurons in PLP–αSyn Mice

**Fig. 2:**
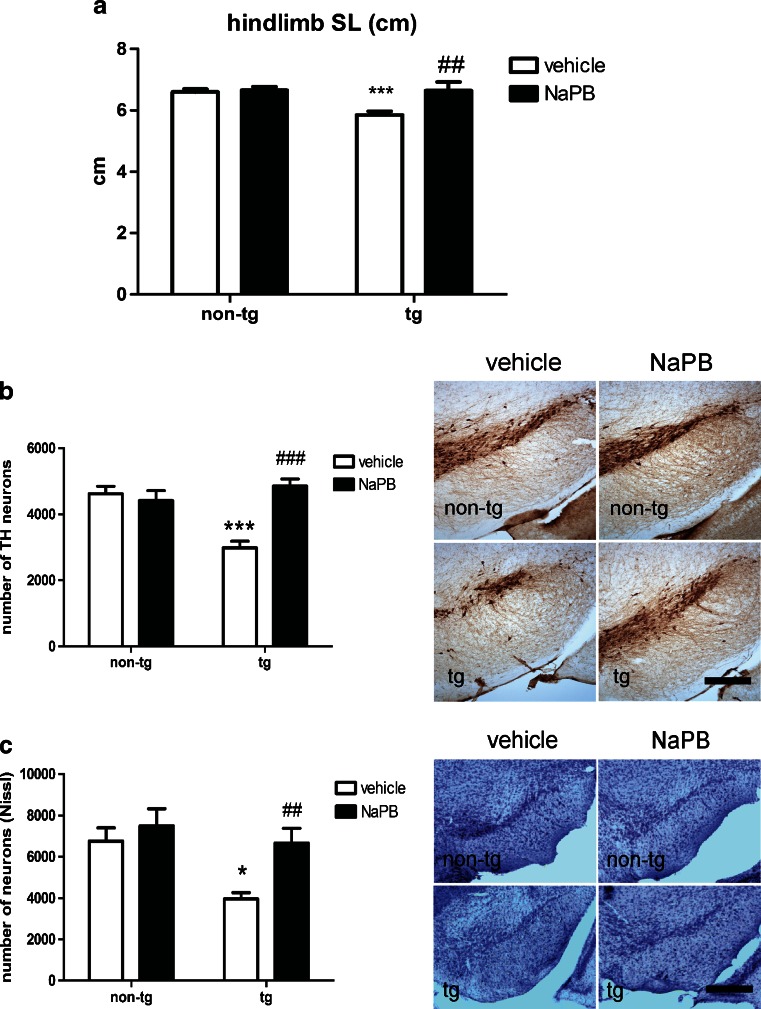
Beneficial effects of sodium phenylbutyrate (NaPB) on motor behavior and nigral neurodegeneration in aged PLP–αSyn mice. (A) Vehicle-treated PLP–αSyn mice (tg; *n*_tg + vehicle_ = 6) > 11 months of age showed significantly reduced stride length (SL) of the hindlimbs compared with vehicle-treated nontransgenic controls (non-tg; *n*_non-tg + vehicle_ = 11; ***p < 0.001). NaPB treatment of tg mice led to a significant improvement of the stride length (^##^ *p* < 0.01) back to the levels of non-tg controls, while stride length was unaffected in non-tg mice receiving NaPB (*n*_tg + NaPB_ = 4; *n*_non-tg + NaPB_ = 9). (B) Tyrosine hydroxylase (TH) immunohistochemistry revealed significant loss of dopaminergic neurons in SNc of tg *versus* non-tg mice receiving vehicle (*n*_tg + vehicle_ = 10; *n*_non-tg + vehicle_ = 10; ***p <0.001). Treatment with NaPB rescued nigral dopaminergic neurons in tg mice (*n*_tg + NaPB_ = 8; ^###^ *p* <0.001), while the treatment had no effect in non-tg animals (*n*_non-tg + NaPB_ = 6). (C) Nissl staining confirmed the observed effects of NaPB treatment as reported with TH immunohistochemistry: there was significant loss of neurons in SNc of tg *versus* non-tg mice receiving vehicle (*n*_tg + vehicle_ = 4; *n*_non-tg + vehicle_ = 8; *p <0.05). Treatment with NaPB rescued nigral neurons in tg mice (*n*_tg + NaPB_ = 6; ^##^ *p* <0.01), while the treatment had no effect in non-tg animals (*n*_non-tg + NaPB_ = 6). Groups were compared by 2-way analysis of variance followed by post-hoc Bonferroni test. Data are presented as mean ± SEM. Scale bars = 500 μm

We found significantly shortened stride length in aged PLP–αSyn transgenic mice (henceforth also called transgenic MSA mice) compared with age-, sex-, and background-matched nontransgenic control mice when recorded at 25 cm/s in the DigiGait system, confirming previous data [12]. Eight weeks of NaPB treatment restored the stride length of the transgenic MSA mice similar to that of the nontransgenic controls (Fig. 2a).

Next we sought to define the number of nigral neurons in mice treated with NaPB as we have previously shown that these transgenic MSA mice are characterized by mild nigral neurodegeneration [12, 13]. Our analysis demonstrated improved cell survival at the level of SNc after NaPB treatment. In the untreated MSA mice, the number of tyrosine hydroxylase (TH)-positive neurons in the SNc was significantly decreased when compared with nontransgenic controls (*p* < 0.001). In animals treated with NaPB, the number of TH-positive neurons in transgenic MSA mice approximated the levels in nontransgenic controls (Fig. 2b). To ensure that the beneficial effect of NaPB in transgenic MSA mice reflects cell survival and does not merely result from changed TH-promoter activity [14], we performed standard Nissl staining on the tissue, which confirmed the findings of TH immunohistochemistry (Fig. 2c).

### NaPB-induced Nigral Neuroprotection is Linked to its HDACi Activity and Reduction of αSyn Inclusions in Transgenic MSA Mice

**Fig. 3:**
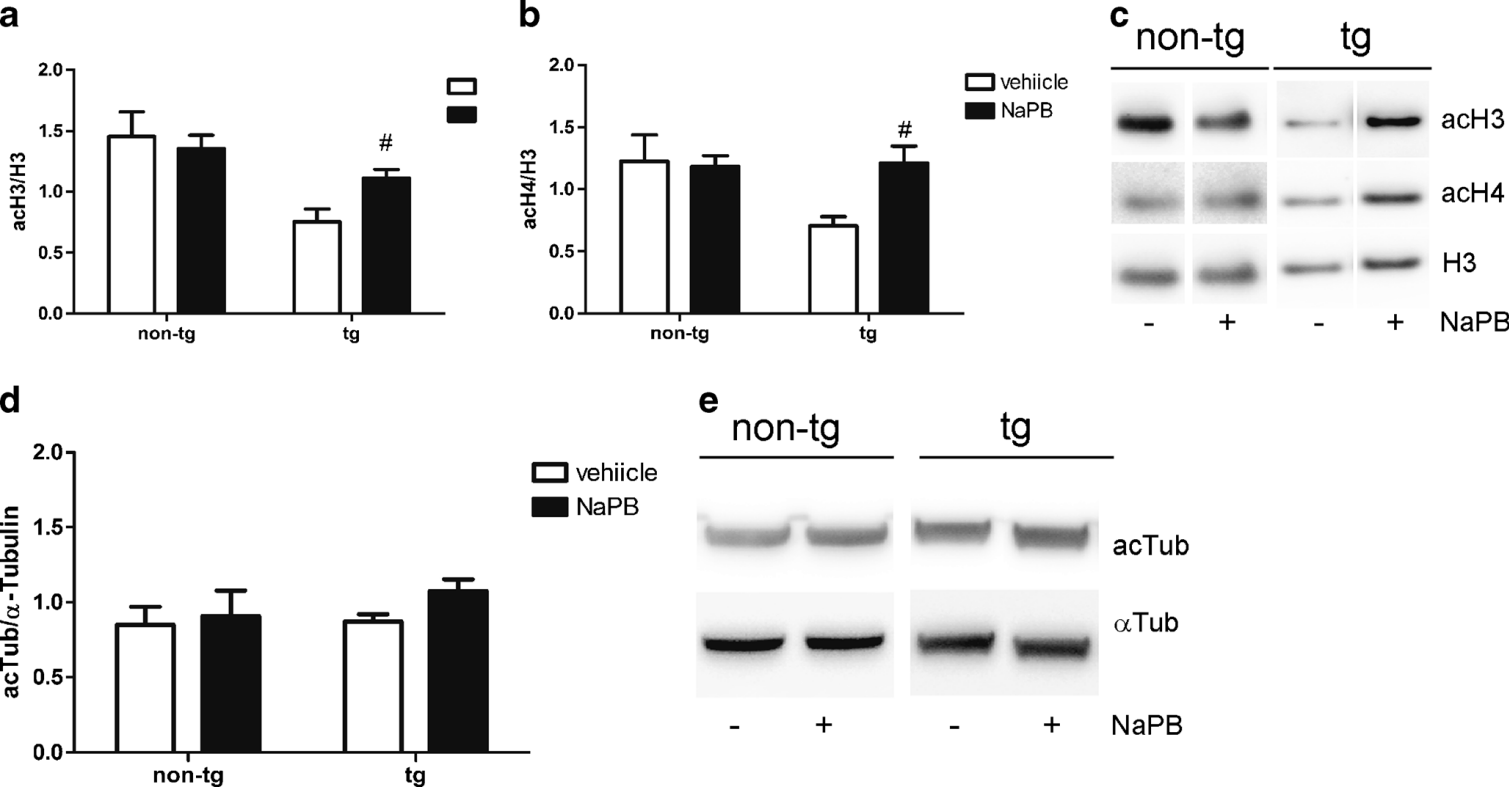
Histone deacetylase inhibitor activity of sodium phenylbutyrate (NaPB) in aged PLP–αSyn mice (tg) and non-transgenic (non-tg) controls. NaPB induced significant increase of (A) H3 acetylation (acH3), and (B) H4 acetylation (acH4) in tg mice but had no effect on the acetylation of histones in non-tg mice. (C) Histone extracts were analyzed by Western blotting and the band intensities for acH3 and acH4 were normalized to the total H3 level. (D) NaPB treatment had no effect on tubulin acetylation (acTub). (E) Radioimmunoprecipitation assay lysates were analyzed by Western blotting and the intensity of acTub bands was normalized to the total α-tubulin (αTub) expression. Treatment groups were compared by *t* test. Data are presented as mean ± SEM (for all groups *n* = 4)

Next, we aimed to identify the mechanisms of neuroprotection by NaPB in the transgenic mouse model of MSA. As a first step to test the HDACi activity of NaPB, we analyzed the levels of histone H3 and H4 acetylation in vehicle- *versus* NaPB-treated mice. Treatment with the nonselective pan-HDACi NaPB significantly increased the level of acH3 and acH4 in MSA mice but had no effect on the histone acetylation in non-PLP–αSyn control mice (Fig. 3a–c).

The cytoskeletal protein α-tubulin represents a nonhistone target for HDACs. Restored acetylation of nonhistone substrates for HDACs that belong to classes I, II, and III can modulate cellular signaling and exert neuroprotective effects [15]. To determine whether cytoskeletal changes linked to α-tubulin acetylation after NaPB treatment occur in the PLP–αSyn mouse, we analyzed the expression levels of acetylated α-tubulin. We detected no significant differences in the acetylation profile of α-tubulin between NaPB- and vehicle-treated mice (Fig. 3d, e). We conclude that changes in the acetylation profile of α-tubulin do not account for the findings.

**Fig. 4:**
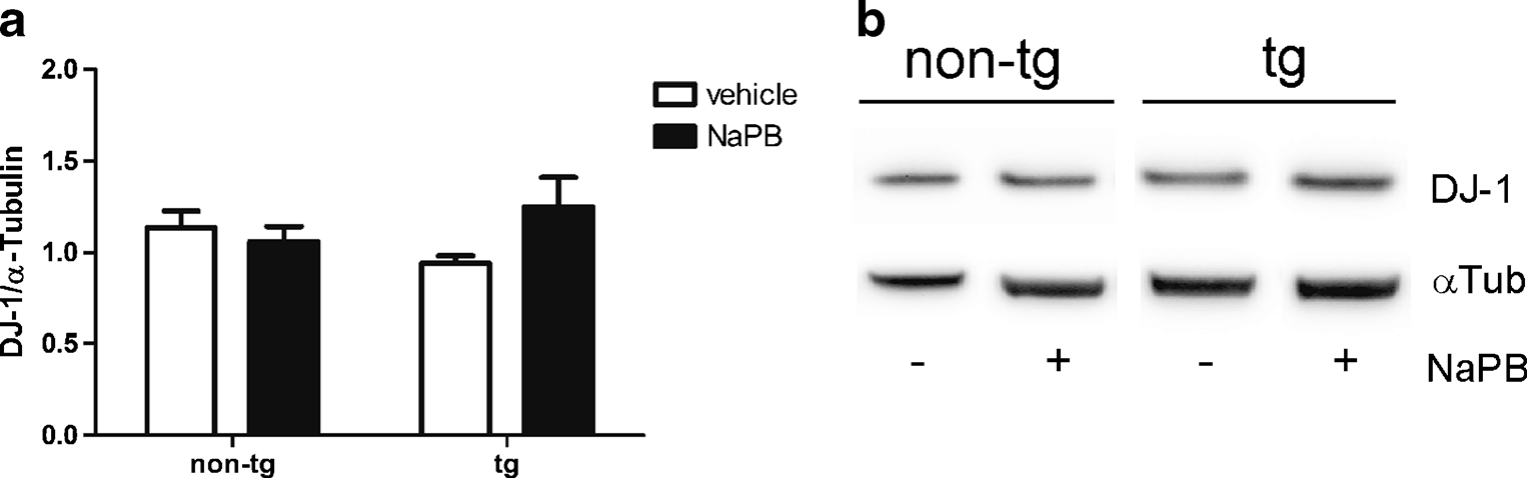
Sodium phenylbutyrate (NaPB) treatment does not affect levels of DJ-1 expression in the midbrain of neither aged PLP–αSyn mice (tg) nor non-tg controls. (A) NaPB had no significant effect of DJ-1 expression in tg and non-tg mice. (B) Radioimmunoprecipitation assay lysates were analyzed by Western blotting and the intensity of DJ-1 bands was normalized to the total α-tubulin (αTub) expression. Groups were compared by *t* test. Data are presented as mean ± SEM (for all groups *n* = 4)

Zhou et al. [16] suggested that treatment with NaPB in animal models of PD may lead to a significant upregulation of the DJ-1 protein linked to nigral neuroprotection. In keeping with this notion, we hypothesized that NaPB upregulates the endogenous antioxidant DJ-1 in the transgenic mouse model of MSA, leading to the observed nigral neuroprotection. To address the question, we measured the levels of DJ-1 protein expression in vehicle- *versus* NaPB-treated animals. We detected no significant changes in the expression levels of DJ-1 between the treatment groups (Fig. 4).

**Fig. 5:**
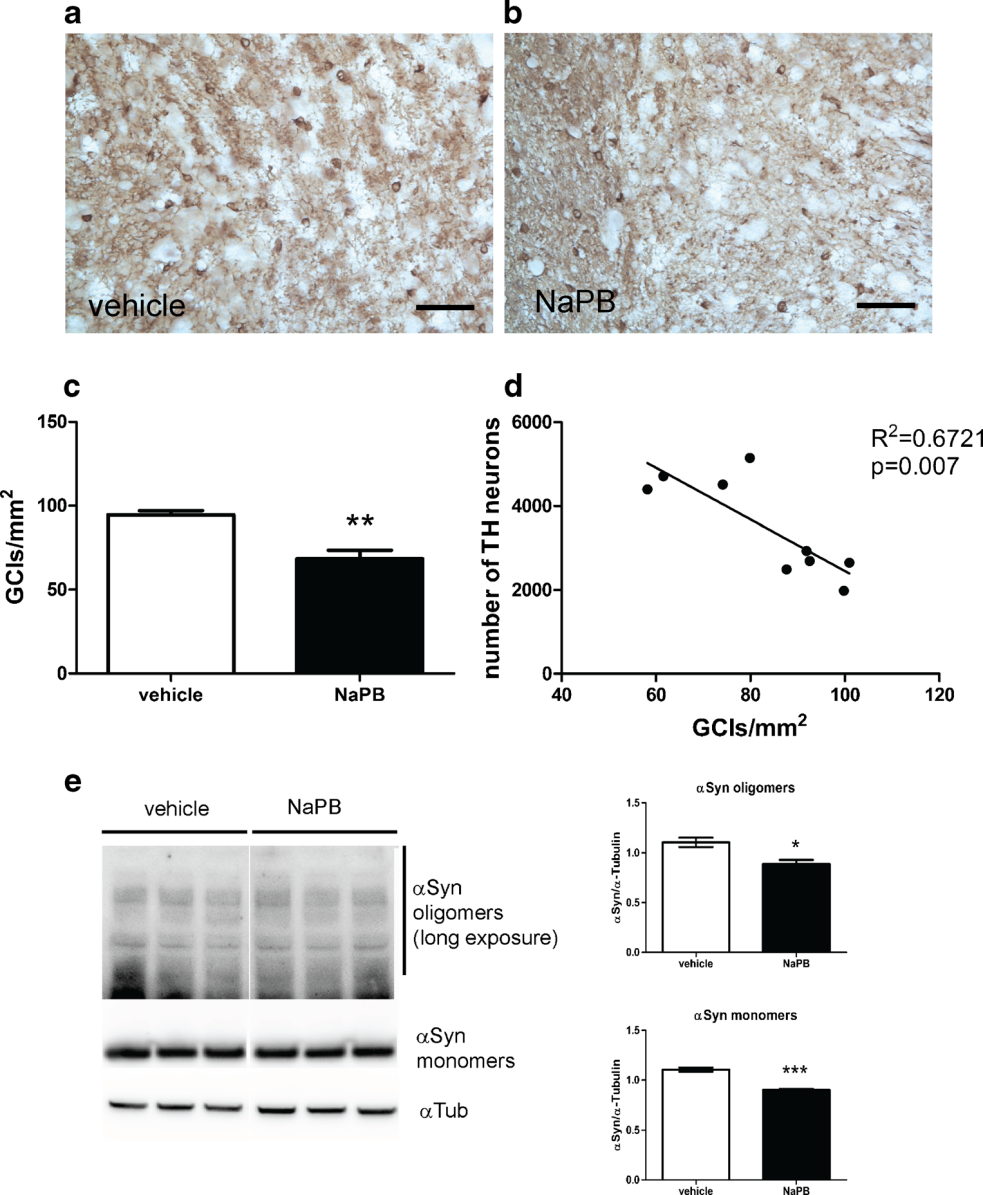
Sodium phenylbutyrate (NaPB) treatment reduces the density of glial cytoplasmic inclusions (GCIs) in the midbrain in aged PLP–αSyn mice. To identify human αSyn-positive GCIs in mice receiving (A) vehicle or (B) NaPB, 15G7 immunohistochemistry was performed. NaPB significantly decreased the density of GCIs in MSA mice when compared with vehicle-treated animals, as shown by *t* test analysis. Data are presented as mean ± SEM, *n*_vehicle_ = 5; *n*_NaPB_ = 4. (C) Linear regression analysis showed significant correlation between the density of GCIs and the number of dopaminergic neurons in substantia nigra pars compacta of MSA mice (D). Western blot analysis of αSyn protein levels in vehicle- and NaPB-treated MSA mice confirmed the significant reduction of the expression of αSyn monomers (***p <0.001) and oligomers (*p <0.05) after NaPB therapy (E). Scale bars = 50 μm. TH = tyrosine hydroxylase

Finally, NaPB could function as a chemical chaperone, thereby decreasing pathological protein aggregation [17]. NaPB treatment in the transgenic mouse model of MSA significantly reduced the density of αSyn-positive GCIs in SNc compared with vehicle-treated animals (Fig. 5a–c). We identified a significant negative correlation between the number of TH-positive neurons and the density of GCIs in SNc of the transgenic MSA mice (Fig. 5d). The effects of NaPB treatment on the levels of αSyn in MSA mice were also confirmed by Western blotting (Fig. 5e).

## Discussion

The current study provides, for the first time, evidence on the beneficial effects of HDACi in aged PLP–αSyn mice. We administered the drug daily for 8 weeks in mice overexpressing human αSyn under the control of the PLP promoter in oligodendrocytes [10], and reflecting GCI-like pathology coupled with motor disability and nigral degeneration. We demonstrated previously that these aged transgenic mice develop shortened stride length compared with age-matched wild-type controls [12]. The present study confirmed these observations and showed that NaPB treatment significantly improved hindlimb stride length. In agreement with the findings on motor improvement, we have shown that NaPB treatment protects nigral dopaminergic neurons in the MSA mouse model. Thirty-five percent of dopaminergic TH-positive neurons were lost in the SNc of PLP–αSyn mice, and this loss was rescued successfully by NaPB treatment. It has been suggested that HDACi are able to upregulate the activity of the TH promoter [14]. It is possible to speculate that the increased number of TH-positive neurons in SNc was actually related to changed expression of TH. We confirmed the neuronal rescue effect of NaPB in SNc of MSA mice in the current experiment by estimation of the total number of neurons in the SNc in classical Nissl staining. In summary, the rescue of nigral neuronal integrity appeared in temporal sequence with gait improvement after treatment with NaPB in PLP–αSyn mice.

Our findings on nigral neuroprotection through NaPB treatment are in concert with observations in different PD models. Roy et al. [18] previously examined the efficacy of NaPB in 1-methyl-4-phenyl-1,2,3,6-tetrahydropyridine (MPTP)-intoxicated C57Bl/6 mice and observed significantly less neurodegeneration in the SNc after treatment. Striatal dopamine was decreased by 76% in MPTP-intoxicated mice, whereas NaPB treatment in MPTP-intoxicated mice limited the decrease to 20–25%. Additionally, the study showed that NaPB improved motor function in the MPTP model of PD [18]. These effects have been repeatedly confirmed in studies examining the neuroprotective effects of NaPB in toxin-induced models of PD, including MPTP toxicity in mice [16], 6-hydroxydopamine toxicity in rats [19], and rotenone-induced nigral degeneration in mice [20].

Several candidate mechanisms, as listed further, have been suggested to contribute to the neuroprotection triggered by NaPB. We analyzed these mechanisms in relation to the nigral neuroprotection in the transgenic PLP–αSyn mouse in order to identify the therapeutic relevance of HDACi to MSA-specific disease events (i.e., GCI-induced nigral degeneration). In response to NaPB treatment in the transgenic MSA mouse we identified increased acetylation levels of the histone core protein H3. The effects of NaPB treatment on H3 acetylation are in line with previous studies. Mice treated with 6-hydroxydopamine showed a general reduction in H3 acetylation that was restored to normal levels after NaPB treatment [19]. In an Alzheimer’s disease mouse model, H3 hyperacetylation upon NaPB treatment was linked to amelioration of the cognitive deficits [11]. The current results suggest that—linked to its HDACi activity—NaPB treatment may interfere with the epigenetic control in the model of MSA-like neurodegeneration and therefore lead to nigral neuroprotection. HDACi achieve histone hyperacetylation enhancing gene transcription, owing to a widened chromatin structure [3]. Therefore, HDACi might be able to reverse specifically toxic effects of αSyn in the nucleus, where the protein has been shown to interact with histones and carry out hypoacetylation [9]. Increased H3 acetylation seems to be involved in the neuroprotective effects of NaPB on MSA pathogenesis; however, it is unclear at this stage, whether neuroprotection is linked to direct epigenetic modifications in the nigral neurons, or rather reflects epigenetic effects in oligodendrocytes that provide improved trophic support for degenerating neurons.

HDACs may interfere with acetylation of cytosolic proteins like α-tubulin; however, NaPB treatment did not affect the acetylation of α-tubulin in the transgenic MSA mouse. Our findings are in concert with previous reports showing that α-tubulin acetylation is carried out by HDAC6 and NAD-dependent deacetylase sirtuin-2, which were not affected by NaPB [21].

Next, we observed a strong reduction of the GCI load that correlated with nigral neuroprotection in NaPB-treated transgenic MSA mice. Therefore, reduction of αSyn inclusion pathology in the brains of MSA mice may be an important factor for the neuroprotection achieved by NaPB treatment. Several other strategies to reduce αSyn aggregation have been implemented recently and support this hypothesis. Immunization has been shown to reduce αSyn inclusions in models of α-synucleinopathies and exert neuroprotection and phenotypic improvement [22]. Small molecular tweezers, such as CLR01, have been proposed to reduce αSyn aggregation linked to neuroprotection and symptom amelioration in models of PD [23]. The mechanism of reduction of αSyn aggregates in response to NaPB treatment is not clear; however, previous studies confirm this effect in various different models [20, 24]. Apart from its epigenetic effects, NaPB may play a role of a chemical chaperone that leads to amelioration of protein aggregation in MSA similar to other disorders [17].

Finally, previous work proposed that NaPB may trigger the activation of endogenous antioxidants and therefore provide neuroprotection [25]. Zhou et al. [16] showed that NaPB treatment can upregulate DJ-1 expression levels in both C57BL/6 mice and the Thy1-Y39C αSyn mouse model of PD, which was further linked to neuroprotection and reduced disease progression [16]. However, we did not observe antioxidant effects of NaPB treatment in aged transgenic MSA mice, as measured by DJ-1 levels in SNc with the dosing regimen applied here (different from the one in the study by Zhou et al. [16]), suggesting that factors other than DJ-1-mediated cell stress responses are pivotal for the neuroprotection reported in the current experiment.

The PLP–αSyn mouse model of MSA has been previously applied in several preclinical studies to screen for disease modification by candidate drugs. In most of the cases the accelerated model of MSA-like neurodegeneration was used, that is, the pathology was modeled by a combination of the transgenic αSyn overexpression and oxidative stress induced by 3-nitropropionic acid [26–29]. This is the first study that tests the efficacy of a drug in the pure transgenic model at a late stage of the disease when functional changes and neurodegeneration linked to the oligodendroglial α-synucleinopathy occur progressively with aging. Several extensive studies have shown the face validity of the PLP–αSyn mouse to replicate functional and neuropathological features of human MSA [10, 12, 13, 30–34]. The current study provides the first proof for a predictive validity of the model by identifying the relationship between the triggering factor (αSyn-positive oligodendroglial inclusions) and the outcome (nigral neurodegeneration and motor deficits) in response to a therapeutic intervention [35].

In conclusion, our data show that HDACi leads to nigral dopaminergic neuroprotection and amelioration of motor impairments in a transgenic mouse model of MSA. We propose that increased H3 acetylation interferes with reduction of nigral GCI density and therefore contributes to the beneficial effects of NaPB. Our results support a potential therapeutic role of HDACi in MSA, as well as in other α-synucleinoapthies such as dementia with Lewy bodies, and PD.

## Electronic supplementary material

Below is the link to the electronic supplementary material.

ESM 1 (PDF 1225 kb)

## References

[1] Fischer A (2014). Targeting histone-modifications in Alzheimer's disease. What is the evidence that this is a promising therapeutic avenue?. Neuropharmacology.

[2] Graff J, Tsai LH (2013). Histone acetylation: molecular mnemonics on the chromatin. Nat Rev Neurosci.

[3] Urdinguio RG, Sanchez-Mut JV, Esteller M (2009). Epigenetic mechanisms in neurological diseases: genes, syndromes, and therapies. Lancet Neurol.

[4] Fanciulli A, Wenning GK (2015). Multiple-system atrophy. N Engl J Med.

[5] Sturm E, Stefanova N (2014). Multiple system atrophy: genetic or epigenetic?. Exp Neurobiol.

[6] Spillantini MG, Goedert M (2000). The alpha-synucleinopathies: Parkinson's disease, dementia with Lewy bodies, and multiple system atrophy. Ann N Y Acad Sci.

[7] Nishie M, Mori F, Yoshimoto M (2004). A quantitative investigation of neuronal cytoplasmic and intranuclear inclusions in the pontine and inferior olivary nuclei in multiple system atrophy. Neuropathol Appl Neurobiol.

[8] Cykowski MD, Coon EA, Powell SZ (2015). Expanding the spectrum of neuronal pathology in multiple system atrophy. Brain.

[9] Kontopoulos E, Parvin JD, Feany MB (2006). Alpha-synuclein acts in the nucleus to inhibit histone acetylation and promote neurotoxicity. Hum Mol Genet.

[10] Kahle PJ, Neumann M, Ozmen L (2002). Hyperphosphorylation and insolubility of alpha-synuclein in transgenic mouse oligodendrocytes. EMBO Rep.

[11] Ricobaraza A, Cuadrado-Tejedor M, Perez-Mediavilla A (2009). Phenylbutyrate ameliorates cognitive deficit and reduces tau pathology in an Alzheimer's disease mouse model. Neuropsychopharmacology.

[12] Stefanova N, Reindl M, Neumann M (2005). Oxidative stress in transgenic mice with oligodendroglial alpha-synuclein overexpression replicates the characteristic neuropathology of multiple system atrophy. Am J Pathol.

[13] Stefanova N, Reindl M, Neumann M (2007). Microglial activation mediates neurodegeneration related to oligodendroglial alpha-synucleinopathy: implications for multiple system atrophy. Mov Disord.

[14] Kim HS, Park JS, Hong SJ (2003). Regulation of the tyrosine hydroxylase gene promoter by histone deacetylase inhibitors. Biochem Biophys Res Commun.

[15] Dietz KC, Casaccia P (2010). HDAC inhibitors and neurodegeneration: at the edge between protection and damage. Pharmacol Res.

[16] Zhou W, Bercury K, Cummiskey J (2011). Phenylbutyrate up-regulates the DJ-1 protein and protects neurons in cell culture and in animal models of Parkinson disease. J Biol Chem.

[17] Winter L, Staszewska I, Mihailovska E (2014). Chemical chaperone ameliorates pathological protein aggregation in plectin-deficient muscle. J Clin Invest.

[18] Roy A, Ghosh A, Jana A (2012). Sodium phenylbutyrate controls neuroinflammatory and antioxidant activities and protects dopaminergic neurons in mouse models of Parkinson's disease. PLoS One.

[19] Sharma S, Taliyan R, Singh S (2015). Beneficial effects of sodium butyrate in 6-OHDA induced neurotoxicity and behavioral abnormalities: modulation of histone deacetylase activity. Behav Brain Res.

[20] Inden M, Kitamura Y, Takeuchi H (2007). Neurodegeneration of mouse nigrostriatal dopaminergic system induced by repeated oral administration of rotenone is prevented by 4-phenylbutyrate, a chemical chaperone. J Neurochem.

[21] Chuang DM, Leng Y, Marinova Z (2009). Multiple roles of HDAC inhibition in neurodegenerative conditions. Trends Neurosci.

[22] Valera E, Spencer B, Masliah E (2016). Immunotherapeutic approaches targeting amyloid-beta, alpha-synuclein, and tau for the treatment of neurodegenerative disorders. Neurotherapeutics.

[23] Attar A, Bitan G (2014). Disrupting self-assembly and toxicity of amyloidogenic protein oligomers by "molecular tweezers"—from the test tube to animal models. Curr Pharm Des.

[24] Ono K, Ikemoto M, Kawarabayashi T (2009). A chemical chaperone, sodium 4-phenylbutyric acid, attenuates the pathogenic potency in human alpha-synuclein A30P + A53T transgenic mice. Parkinsonism Relat Disord.

[25] Chan JY, Chan SH (2015). Activation of endogenous antioxidants as a common therapeutic strategy against cancer, neurodegeneration and cardiovascular diseases: A lesson learnt from DJ-1. Pharmacol Ther.

[26] Stefanova N, Poewe W, Wenning GK (2008). Rasagiline is neuroprotective in a transgenic model of multiple system atrophy. Exp Neurol.

[27] Stefanova N, Georgievska B, Eriksson H (2012). Myeloperoxidase inhibition ameliorates multiple system atrophy-like degeneration in a transgenic mouse model. Neurotox Res.

[28] Kaindlstorfer C, Sommer P, Georgievska B (2015). Failure of neuroprotection despite microglial suppression by delayed-start myeloperoxidase inhibition in a model of advanced multiple system atrophy: clinical implications. Neurotox Res.

[29] Fellner L, Kuzdas-Wood D, Levin J (2016). Anle138b partly ameliorates motor deficits despite failure of neuroprotection in a model of advanced multiple system atrophy. Front Neurosci.

[30] Stemberger S, Poewe W, Wenning GK (2010). Targeted overexpression of human alpha-synuclein in oligodendroglia induces lesions linked to MSA-like progressive autonomic failure. Exp Neurol.

[31] Krismer F, Wenning GK, Li Y (2013). Intact olfaction in a mouse model of multiple system atrophy. PLoS One.

[32] Boudes M, Uvin P, Pinto S (2013). Bladder dysfunction in a transgenic mouse model of multiple system atrophy. Mov Disord.

[33] Kuzdas D, Stemberger S, Gaburro S (2013). Oligodendroglial alpha-synucleinopathy and MSA-like cardiovascular autonomic failure: experimental evidence. Exp Neurol.

[34] Stefanova N, Wenning GK (2015). Animal models of multiple system atrophy. Clin Auton Res.

[35] Belzung C, Lemoine M (2011). Criteria of validity for animal models of psychiatric disorders: focus on anxiety disorders and depression. Biol Mood Anxiety Disord.

